# The lung-brain axis in multiple sclerosis: Mechanistic insights and future directions

**DOI:** 10.1016/j.bbih.2024.100787

**Published:** 2024-05-03

**Authors:** Lara Kular

**Affiliations:** Department of Clinical Neuroscience, Karolinska Institutet, Center for Molecular Medicine, Karolinska University Hospital, Stockholm, Sweden

**Keywords:** Multiple sclerosis, Lung-brain axis, Smoking, Immune, Epigenetics

## Abstract

Multiple sclerosis is a chronic inflammatory demyelinating disease of the central nervous system with progressive lifelong disability. Current treatments are particularly effective at the early inflammatory stage of the disease but associate with safety concerns such as increased risk of infection. While clinical and epidemiological evidence strongly support the role of a bidirectional communication between the lung and the brain in MS in influencing disease risk and severity, the exact processes underlying such relationship appear complex and not fully understood. This short review aims to summarize key findings and future perspectives that might provide new insights into the mechanisms underpinning the lung-brain axis in MS.

## 1 Introduction

Multiple sclerosis is a leading cause of lifelong disability in young and middle-aged adults, particularly women. Disease pathology is characterized by infiltration of immune cells into the central nervous system (CNS), autoimmune demyelination and subsequent neuro-axonal loss (Jakimovski et al., 2023). Although the exact cause of MS is unknown, this highly heterogeneous disease is triggered by environmental factors such as Epstein–Barr virus (EBV) infection, smoking, low vitamin D and obesity, in genetically predisposed individuals. The genetic risk is dominated by the HLA-DRB1*15:01 allele involved in antigen presentation to T cells and overall implicates deregulation of peripheral and CNS-resident immune cells (International Multiple Sclerosis Genetics and C., 2019). Due to a remarkable advance in the field, current treatments are now highly effective during the inflammatory-active relapsing-remitting stage, although they broadly target the peripheral immune system and therefore associate with safety concerns such as increased risk of infections (Jakimovski et al., 2023). People with MS (pwMS) will eventually transition to a distinct progressive stage characterized by CNS-confined inflammation and neurodegeneration, leading to continuous worsening of disability. Recent advance in the genetic mapping of variants influencing disease severity underscored impairment of CNS cells in disease progression (International Multiple Sclerosis Genetics et al., 2023). Yet, the lack of beneficial treatments for progressive MS, likely due to our still partial understanding of disease processes, remains the greatest challenge in the care of patients with MS. While clinical and epidemiological evidence support a bidirectional communication between the lung and the brain in MS - inflammation in one organ can affect the other and *vice versa,* the exact processes underlying such relationship appear complex and not fully understood. This short review aims to summarize key findings and future perspectives that might aid in our understanding of the mechanisms underpinning the lung-brain axis in MS.

### 1.1 Clinical and epidemiological evidence of a bi-directional lung-brain relationship in MS

As briefly recapitulated in this section, epidemiological studies have established a dynamic interaction between inflammation of the lungs and MS susceptibility and severity.

#### 1.1.1 Smoking and other environmental exposures

The strongest evidence of the impact of the lungs’ integrity on MS disease is coming from studies examining the effect of cigarette smoking (comprehensively reviewed by Rosso and Chitnis (Rosso et al., 2020)). Both active and passive smoking has been unanimously associated with an elevated risk of developing inflammatory and autoimmune diseases such as MS, particularly in genetic susceptible individuals, i.e. this risk being drastically increased in carriers of the major MS *HLA* risk variants (Hedstrom et al., 2011a, 2011b). Importantly, smoke exposure also strongly favors a more active and severe disease with faster disease progression, lesion load and brain atrophy, increasing the disability burden (physical, psychological and cognitive) experienced by pwMS (Rosso et al., 2020; Ramanujam et al., 2015; Wu et al., 2023; Hernan et al., 2005; Healy et al., 2009; Manouchehrinia et al., 2013; Zivadinov et al., 2009). Interestingly, the effect of smoking on MS on brain atrophy can occur at an early stage of MS (Rosso et al., 2020; Graetz et al., 2019). The effect of smoking on MS risk and progression follows a dose-response association according to the duration and intensity of smoking, which further support causality (Rosso et al., 2020; Hedstrom et al., 2009, 2011a; Hernan et al., 2005). Overall, the impact of smoking is likely mediated by smoking-related lung irritation and inflammation (described in the next section) rather than nicotine itself as the use of oral tobacco does not predispose for MS risk or progression (Wu et al., 2023; Hedstrom et al., 2009). Additionally, exposure to specific air pollutants has been linked to an elevated risk of developing MS as well (Hedstrom et al., 2023; Tang et al., 2021; Tateo et al., 2019; Ziaei et al., 2022) although discrepancies between studies, with sometimes conflicting results (Bai et al., 2018; Chen et al., 2017a; Abbaszadeh et al., 2021), warrant further confirmation.

While exposure to virus, particularly infection with EBV, are well-recognized risk factors for MS, other infections have been associated with disease exacerbation. Among them, common viral upper respiratory tract infections, such as with influenza, have been linked to an increased relapse rate (Marrodan et al., 2019). On the contrary, studies examining the effect of COVID-19 infection found no evidence of disease exacerbation in pwMS infected by SARS-CoV-2 virus (Seyedmirzaei et al., 2024; Aghajanian et al., 2024). While the risk of contracting COVID-19 itself was not higher in pwMS, some (but not all) studies report a higher risk of hospitalization after infection in pwMS compared to the general population (Barzegar et al., 2023; Moghadasi et al., 2021). This association is likely influenced by the presence of comorbidities (Garcia-Dominguez et al., 2024; Bazylewicz et al., 2023).

#### 1.1.2 Comorbid lung disorders

People diagnosed with MS are more likely to develop comorbid conditions, particularly psychiatric cardiovascular, respiratory and inflammatory disorders (Magyari and Sorensen, 2020). Among them, asthma and chronic obstructive pulmonary disease (COPD) are more frequent in young and middle-aged pwMS compared to matched control population (Hill et al., 2019; Marrie et al., 2016). While the exact relationship between MS and asthma remains to be fully ascertained (Ghoshouni et al., 2024), a diagnosis of COPD has been further associated with higher MS risk and more severe disease course (Egesten et al., 2008; Conway et al., 2017) and conversely, pwMS are more likely to develop COPD (Ghoshouni et al., 2024). Yet, one cannot exclude the potential influence of other concomitant comorbidities, such as hypertension, in the association between chronic lung diseases and MS disease severity (Ghoshouni et al., 2024; Sorensen et al., 2021). In line with this, given the unequivocal negative impact of smoking on cardiovascular and respiratory functions, smokers living with MS present with an elevated comorbidity burden and worsened disease outcomes (Rosso et al., 2020). The synergetic multifaceted and dynamic nature of the comorbidities, including respiratory, contributing to MS disease severity and progression warrant further investigation. On the other hand, compelling evidence supports respiratory dysfunction in pwMS as secondary to enduring long-standing disease. Indeed, pulmonary function, measured by dynamic spirometry, is typically affected at advanced and progressive stages of the disease (Tzelepis et al., 2015). Respiratory complications also significantly contribute to MS mortality, with lung infections standing among the leading causes of death in the MS population compared to matched control individuals (Harding et al., 2020; Kingwell et al., 2020; Fedeli et al., 2023; Hirst et al., 2008). While respiratory insufficiency is generally a characteristic of long-term chronic disease with high disability and comorbidities, additional evidence suggests that alteration of the lung function can occur earlier during MS disease development. Accordingly, reduced respiratory functional capacity, likely due to early respiratory muscle weakness, can emerge at initial phases of disease development and precede complications later (Tzelepis et al., 2015).

Collectively, the outcomes of clinical and epidemiological studies converge to an intricate link between the lungs and the CNS in MS. Irritation and/or inflammation of the lung increases the susceptibility and severity of MS disease, and in turn, established CNS autoimmunity and ensuing damage enhance the lungs vulnerability to dysfunction.

### 1.2 Insights into the potential mechanisms underpinning the lung-brain axis in MS

The exact mechanisms underlying the lung-brain axis in MS remain largely unknown, but outcomes of case-control cohorts and experimental model of MS, summarized in this section, imply the contribution of both direct and indirect pathways between the lung and the CNS.

#### 1.2.1 Immune cell mediation of the crosstalk between the lung and the CNS

A growing body of evidence supports the communication between the lung and the brain to be mediated at least partly by immune cells. As described in the case of other autoimmune diseases, irritation or inflammation of the lungs, induced for example by smoke exposure, can have immunological consequences by creating an inflammatory shift towards damaged and pro-inflammatory local immune responses and by stimulating autoreactive lymphocytes (Rosso et al., 2020).

Findings established in the rodent MS-like disease model, experimental autoimmune encephalomyelitis (EAE), present the lungs as an inflammatory niche where CNS-specific autoreactive T cells gain the capacity to infiltrate the CNS prior to disease onset (Odoardi et al., 2012; Glenn et al., 2017, 2019; Blackmore et al., 2017; Chen et al., 2017b). The seminal study from Odoardi et al. (2012) demonstrated that CNS-specific autoreactive T cells become licensed in the lung where they undergo a profound phenotypic reprogramming towards dampened proliferative and activation capacity and enhanced pro-migratory potential. These newly acquired properties further equip T cells with potent chemotactic capability to infiltrate the CNS and induce damage (Odoardi et al., 2012). Other studies further illuminated some of the potential mechanisms underpinning the relationship between upper tract infection and CNS autoimmunity by using a comorbid experimental model where mice were intranasally inoculated with influenza A virus prior or after EAE induction (Glenn et al., 2017, 2019; Blackmore et al., 2017; Chen et al., 2017b). Upper-respiratory influenza infection could trigger mild EAE symptoms in the autoimmune-prone T-cell receptor transgenic 2D2 mice (Blackmore et al., 2017). Moreover, the sole inoculation of wildtype mice with influenza caused time-dependent transcriptomic changes in the CNS indicative of reactive gliosis and IFN signaling (Blackmore et al., 2017). This was consistent with increased trafficking of lymphocytes, monocytes and MHC class II-expressing B cells in the brain of influenza-infected mice compared to control animals (Blackmore et al., 2017). Interestingly, mice that were infected with influenza virus 50 days prior to EAE induction developed an extended and exacerbated EAE disease compared to EAE-only control animals (Chen et al., 2017b). Disease exacerbation was mediated by a change of the lung inflammatory environment before EAE onset that promoted accumulation of CCR5-expressing Th1 CD4^+^ T cells in the lung tissue prior to infiltration into the CNS during EAE clinical course. Administration of CCR5 antagonist could attenuate the EAE disease severity in post-influenza mice (Chen et al., 2017b). Conversely, EAE mice that were subsequently inoculated with influenza showed drastic increase in morbidity and mortality compared to EAE-only, influenza-only or non-CNS autoimmune animals (Glenn et al., 2017). This elevated mortality was not attributed to increased EAE severity but, instead, to a drastic alteration of the lung milieu, with noticeable lung pathology, suppressed effector immune cells (NK and CD8^+^ T cells) activation and subsequent failure to control viral replication. Impaired viral control was further found to be likely caused by the mobilization of phenotypically distinct monocyte-derived myeloid cells recruited to the lungs and suppressing effector cell activation in co-afflicted mice (Glenn et al., 2017). Interestingly, such EAE-dependent myeloid-derived suppressor cells could concomitantly promote the encephalitogenic Th17 polarization of CD4^+^ T cells (Glenn et al., 2019). Thus, in experimental model of MS disease, alterations of the lungs interact with the disease by establishing an inflammatory environment in the lung that is prone to priming of auto-aggressive T cells towards CNS infiltration. Nevertheless, such effect is not specific to the lung or CNS autoimmunity as immune modulation by other mucosal tissues, such as the intestinal barrier, and in other autoimmune diseases has been demonstrated (Rosso et al., 2020). Moreover, additional mechanisms found to further enhance autoimmune reactions involve the presentation of foreign or novel antigens by damaged alveolar macrophages to T cells and a subsequent cross-reactivity to CNS self-antigens. While this mode of action seems particularly relevant in the context of *HLA* class II genetic susceptibility, as described for other autoimmune disorders (Rosso et al., 2020), it remains poorly understood in the case of EAE or MS. Additionally, given the imperfect parallelism between the rodent EAE and human MS diseases, future work is required to confirm these findings in pwMS.

Smoking can exert long-lasting effects through epigenetic mechanisms, and we have demonstrated that DNA methylation (the covalent addition of a methyl group to the CpG cytosines), which regulates gene expression and genome stability, plays a role in disease etiology (Kular et al., 2018; Xavier et al., 2023). By profiling the methylome and transcriptome of bronchoalveolar lavage (BAL) and blood cells in pwMS and healthy donors, we found that smoking interacts with the disease at the molecular level of known smoking-related genes in a dose-dependent manner (Marabita et al., 2017). Indeed, the pack-years measure of smoking load was associated with more pronounced hypomethylation at, for example, the aryl-hydrocarbon receptor repressor (*AHRR*) gene in pwMS in comparison to healthy controls. The typical smoking-induced epigenetic signature could be observed in the primary exposed BAL cells of both pwMS and controls as well, notably with greater effect size compared to the blood compartment (Ringh et al., 2021). Surprisingly, smoking resulted in an additional distinct neuronal-neurodegenerative pattern with affected genes linked to axonal guidance and synaptic (glutamatergic, cholinergic) transmission, in BAL cells of pwMS specifically (Ringh et al., 2021). While the altered pathways overlap with the molecular changes detected in MS neurons post-mortem, many of them have been involved in immune processes outside of the CNS as well (Ringh et al., 2021). Additionally, analyses of non-smokers suggest that BAL cells from pwMS displayed moderate but consistent changes reflecting reduced transcriptional/translational processes and enhanced migratory abilities, compared to healthy controls. Thus, these studies imply that the lung macrophages of pwMS display distinct molecular changes compared to healthy individuals, both in the absence and presence of smoke exposure.

#### 1.2.2 Direct action of lung alterations on the CNS

The direct action of cigarette smoke on the CNS integrity has been suggested in the context of MS (Rosso et al., 2020). Indeed, following smoke exposure, toxic compounds, such as reactive oxygen species, released in the blood circulation can show neurotoxicity as well and as such capable of damaging the neurons and glia involved in the CNS vulnerability to neuroinflammatory insults. A recent study by Hosang et al. (2022) suggested an intriguing link between the lung microbiome and CNS-resident immune cells reactivity in EAE. Neomycin-induced lung dysbiosis characterized by lipopolysaccharide (LPS)-enriched phyla associated with lower susceptibility to develop EAE. The mechanisms did not involve peripheral immune cell deregulation but instead a direct action of LPS-derived circulating factors on the CNS, particularly microglia, leading to reduced proinflammatory response, peripheral immune cell recruitment and clinical signs (Hosang et al., 2022). While this finding warrant replication, this study showed for the first time that the lung microbiome-derived metabolite can regulate CNS autoimmunity by acting in the target organ *in situ* and altering the CNS-resident immune cells inflammatory potential.

**Fig. 1:**
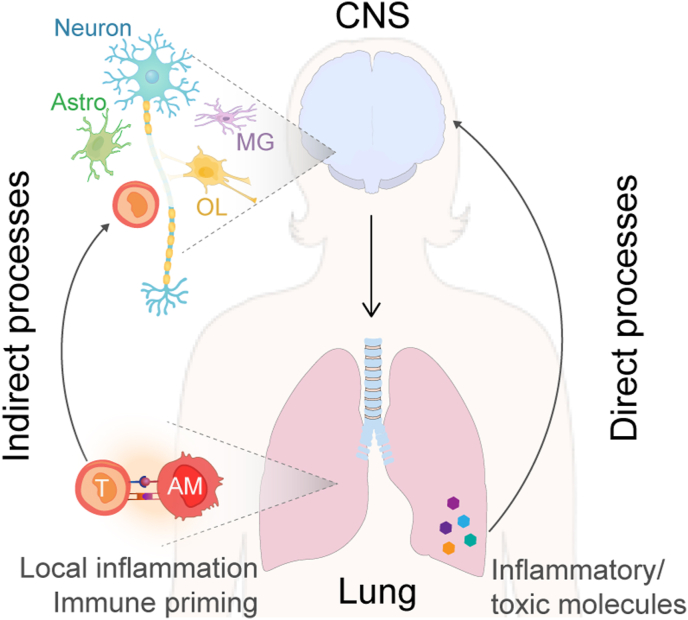
Schematic summary of the processes underlying the bi-directional lung-brain crosstalk in MS. T: T cells, AM: alveolar macrophage, MG: microglia, OL: oligodendrocytes, Astro: astrocyte, CNS: central nervous system.

These findings jointly reinforce the privileged and intertwined relationship between local, peripheral and central immunity in the context of MS, some of these processes might be partly mediated by epigenetic mechanisms. Accordingly, as summarized in Fig. 1, CNS autoimmunity involves not only a prerequisite priming of CNS-specific autoreactive T cells in the lung prior to CNS damage, but also a conversion of local immunity potentially exacerbating the lung immune resilience to respiratory infection. Undoubtedly, additional direct processes, such as via the action of lung-derived molecules on CNS cells and alteration of descending nerve impulses, are superimposed on immune cell mediation and partake in the lung-brain crosstalk in MS.

### 1.3 Future directions in unravelling the processes underpinning the lung-brain axis in MS

This section aims to provide speculative insights into potential future avenues in the research field.

#### 1.3.1 Disentangling the interaction between smoking-related lung inflammation and other risk factors

While each risk factor, such as HLA-DRB1*15:01 and smoking, confer modest effect on MS susceptibility, they interact and drastically increase disease risk in synergy (Olsson et al., 2017). Gene-environment interactions are visible globally at the molecular level with multi-omic integration analysis revealing co-regulated and interconnected networks of genes affected by both genetic and environmental influences (Badam et al., 2021). In line with this, the impact of smoking on blood DNA methylation displayed greater amplitude of changes in a cohort selected as of particular risk, i.e. solely composed of women carriers HLA-DRB1*15:01 and non-carriers of the protective *HLA-A2* variant (Marabita et al., 2017). Although this finding needs to be interpreted with caution given the plausible effect of additional confounders, it implies an interaction of smoking and HLA risk variants at the epigenetic level. This may further impact the lung immunity as suggested by the HLA-dependent differences of global lung macrophage response in smokers (Ockinger et al., 2016). Further synergy may operate in concert with EBV insofar as smoking significantly enhances the association between high anti-EBNA titer and elevated MS risk, this interaction being likely conditioned by age (Simon et al., 2010; Salzer et al., 2014). Consistent with this, smoking has been generally linked with oral EBV loads, EBNA1 seropositivity and EBV reactivation in a dose-dependent manner (He et al., 2019; Hu et al., 2019). Smoke exposure also induces an increase in blood (class-switched) memory B cells (Brandsma et al., 2009), with the yet-to-be proven possibility that smoke-induced lung tissue damage might also unveil neo-antigens as established in other autoimmune conditions. Given that sizeable B cell-specific epigenetic changes characterize MS, smoking and EBV infection (Ewing et al., 2019; Ma et al., 2021; Su et al., 2016), one cannot exclude an epigenetic mediation of the impact of smoking on B cell activation and EBV control as well. Overall, deciphering the exact contribution of smoking-induced changes in concert with *HLA* class II risk variants and EBV control on disease pathogenesis will further aid in our understanding of disease mechanisms towards precision medicine and personalized care.

#### 1.3.2 Deciphering the link between lung inflammation, immunosenescence and MS

A growing body of evidence supports a link between smoking, biological age acceleration including immunosenescence and MS. The smoking-dependent neuronal-like epigenetic signature identified in BAL cells of pwMS mirrors the changes observed in patients at advanced progressive stage of MS (Ewing et al., 2019; Campagna et al., 2022). Interestingly, this pattern was typically found in aging blood immune cells as well (Acevedo et al., 2015; Wang et al., 2018). Chronological age is a known to be associated with a greater risk of transitioning to a progressive MS stage, irrespective of the initial disease course or relapse history (Confavreux and Vukusic, 2006; Scalfari et al., 2011; Tutuncu et al., 2013), and biological age acceleration has emerged as a critical factor in the development and progression of MS (Zhang et al., 2023). Moreover, independent studies exploiting epigenetic clocks as robust predictors of biological age indicated that BAL cells (Klose et al., 2023), peripheral blood immune cells (particularly B cells) (Maltby et al., 2023; Theodoropoulou et al., 2019) and glial cells (Kular et al., 2022) of pwMS exhibit faster aging compared to controls. Importantly, age acceleration was conditioned by the smoking status in the lung and blood compartments (Klose et al., 2023; Maltby et al., 2023; Theodoropoulou et al., 2019) and was likely influenced by a senescence-associated alteration of alveolar macrophage composition in the lung (De Man et al., 2023). Such premature senescence is likely to impact immune function and CNS resilience in MS and understanding the mechanisms behind lung irritation and accelerated aging may assist clinicians in articulating the benefit of smoking cessation to patients.

#### 1.3.3 Refining the exact pathogenic immune cell signature in the inflamed lung in MS

The precise signature of the discrete pathogenic cells driving MS pathogenesis remains unresolved. This is likely due to their low abundance in conventionally studied tissue, such as the blood compartment, and further hamper the development of targeted therapeutic strategies. The specific signature of lung-circulating encephalitogenic T cells in MS-like EAE disease (see previous section) have undeniably illuminated some of the lung-acquired molecular properties prior to CNS damage in rodents. The use of advanced methodologies such as single-cell and/or spatially-deconvoluted profiling of the lung milieu in pwMS has the potential to further refine and specify the cellular culprits and molecular changes driving the human disease. Such approach has revealed distinct changes in the composition of myeloid cellular compartment upon smoking, i.e. with increased proportions of stressed/damaged classical alveolar macrophages and recently recruited monocyte-derived AMs with an activated phenotype (Liegeois et al., 2022). Comparison with profiles of pathogenic immune cells enriched in the cerebrospinal fluid might aid even more in discriminating the causal features of these changes.

## 2 Conclusions

While the field of lung-brain axis in MS is still at its infancy, accumulating evidence supports a bi-directional link between alterations of the lung and the 10.13039/100000144CNS during disease development and progression. The processes underpinning this intertwined relationship partly rely on immune mediation of tissue resilience along the lung-brain axis - inflammatory damage in one organ can influence the vulnerability of the other one, notably via circulating immune cells and inflammatory mediators. Additional direct crosstalk implicates the impact of demyelination of the CNS, notably in breathing control centers, on respiratory function visible at early stage of disease development and responsible for serious respiratory complications at more advanced stages of the disease. Future studies aiming at disentangling the complex interplay between smoking and other factors influencing the risk (HLA variants, EBV infection) and severity (immunosenescence) of MS disease and further resolving the exact smoking-associated molecular architecture and cellular culprits driving disease pathogenesis have the potential to greatly aid in our understanding of disease processes and improve precision medicine.

## CRediT authorship contribution statement

**Lara Kular:** Writing – original draft, Conceptualization.

## Declaration of competing interest

The author declares that they have no known competing financial interests or personal relationships that could have appeared to influence the work reported in this paper.

## Data Availability

No data was used for the research described in the article.
